# Photophobia is associated with lower sleep quality in individuals with migraine: results from the American Registry for Migraine Research (ARMR)

**DOI:** 10.1186/s10194-024-01756-9

**Published:** 2024-04-12

**Authors:** Nina Sharp, Mark J Burish, Kathleen B Digre, Jessica Ailani, Mahya Fani, Sophia Lamp, Todd J. Schwedt

**Affiliations:** 1 The Design School, Arizona State University, Tempe, AZ USA; 2 Department of Neurosurgery, Medical School, The University of Texas Health Science Center at Houston, McGovern, Houston, TX USA; 3 Department of Ophthalmology and Visual Sciences, Department of Neurology, John A Moran Eye Center, University of Utah Health Sciences Center, Salt Lake City, UT USA; 4 Department of Neurology, Medstar Georgetown University Hospital, Washington, DC USA; 5 Psychology Department, Arizona State University, Tempe, AZ USA; 6 Neurology Department, Mayo Clinic Arizona, Phoenix, AZ USA

**Keywords:** Migraine, Headache, Photophobia, Sleep, Photophilia, Light, Circadian

## Abstract

**Background:**

Patients with migraine often have poor sleep quality between and during migraine attacks. Furthermore, extensive research has identified photophobia as the most common and most bothersome symptom in individuals with migraine, second only to headache. Seeking the comfort of darkness is a common strategy for managing pain during an attack and preventing its recurrence between episodes. Given the well-established effects of daily light exposure on circadian activity rhythms and sleep quality, this study aimed to investigate the relationship between photophobia symptoms and sleep quality in a cohort of patients with migraine.

**Methods:**

A cross-sectional observational study was conducted using existing data extracted from the American Registry for Migraine Research (ARMR). Participants with a migraine diagnosis who had completed the baseline questionnaires (Photosensitivity Assessment Questionnaire (PAQ), Generalized Anxiety Disorder-7 (GAD-7), Patient Health Questionnaire-2 (PHQ-2)), and selected questions of the ARMR Sleep questionnaire were included. Models were created to describe the relationship of photophobia and photophilia with various sleep facets, including sleep quality (SQ), sleep disturbance (SDis), sleep onset latency (SOL), sleep-related impairments (SRI), and insomnia. Each model was controlled for age, sex, headache frequency, anxiety, and depression.

**Results:**

A total of 852 patients meeting the inclusion criteria were included in the analysis (mean age (SD) = 49.8 (13.9), 86.6% (*n* = 738) female). Those with photophobia exhibited significantly poorer sleep quality compared to patients without photophobia (*p* < 0.001). Photophobia scores were associated with SQ (*p* < 0.001), SDis (*p* < 0.001), SOL (*p* = 0.011), SRI (*p* = 0.020), and insomnia (*p* = 0.005) after controlling for age, sex, headache frequency, depression, and anxiety, signifying that higher levels of photophobia were associated with worse sleep-related outcomes. Conversely, photophilia scores were associated with better sleep-related outcomes for SQ (*p* < 0.007), SOL (*p* = 0.010), and insomnia (*p* = 0.014).

**Conclusion:**

Results suggest that photophobia is a significant predictor of poor sleep quality and sleep disturbances in migraine. These results underscore the necessity for comprehensive and systematic investigations into the intricate interplay between photophobia and sleep to enhance our understanding and develop tailored solutions for individuals with migraine.

## Background

A migraine attack consists of moderate-to-severe intensity throbbing headache accompanied by a combination of sensory hypersensitivities (e.g., photosensitivity, phonosensitivity, olfactory hypersensitivity, cutaneous allodynia), nausea, and vomiting [1, 2]. Photophobia, defined here as discomfort caused by light and exacerbation of headache by light, is exceedingly prevalent during migraine attacks and can persist, typically to a lesser extent, between attacks [3, 4]. Moreover, numerous migraine studies reported photophobia as the most common, most bothersome symptom other than headache [5–8]. Generalized photophobia (i.e., both ictally and interictally) has been linked to several aspects of migraine: higher generalized photophobia is more common in migraine with aura (MwA) compared to those without aura (MwoA) [9] and higher photophobia scores are associated with greater work-related disability and activity impairment [10].

Individuals with migraine also often report poor sleep quality and sleep disturbances, both as triggers of migraine attacks and as a consequence of the attacks [11]. These disturbances manifest as difficulty falling asleep, staying asleep, self-reported decreased sleep duration, extreme daytime sleepiness, and lack of feeling refreshed after sleep [12–16]. Despite substantial growth in the literature over the past two decades, the precise nature and direction of the relationship between sleep and migraine remains elusive. It is conceivable that sleep disturbances are caused by an ongoing migraine attack or sleep disruption may trigger migraine attacks.

There are multiple lines of evidence to suggest that photophobia and sleep might be related in migraine. Clinically, in two open-label studies green light improves not only headaches but also photophobia and sleep [17, 18], though these data are confounded by the fact that the headaches also improved. Mechanistically, photophobia and light may be related through the circadian system. Light, especially bright light, is the strongest zeitgeber (an environmental cue which resynchronizes our internal biological clock to the external world), and the circadian system regulates the sleep-wake cycle [19]. Specifically, light received through signals from the intrinsically photosensitive retinal ganglion cells (ipRGCs) synchronizes the rhythmic activity of the central clock in the hypothalamus, the suprachiasmatic nuclei (SCN). There are several lines of evidence that migraine has circadian properties, including lower melatonin levels amongst those with migraine and a circadian pattern of attacks; moreover, a variation in a core circadian gene – *CK1δ* –causes both migraine and a circadian rhythm disorder (advanced sleep phase) [20, 21]. Epidemiologically, the Korean Sleep-Headache Study performed latent class analysis and ultimately subtyped patients into 3 groups, one of which was an ictal photophobia/phonophobia group [22]. This photophobia/phonophobia group had the highest proportion of patients with excessive daytime sleepiness on the Epworth Sleepiness Scale, though the findings were not significant (*p* = 0.13) possibly due to the relatively modest enrollment (*n* = 125 across 3 groups). However, an even smaller study (*n* = 48) comparing migraine patients with interictal photophobia, did find a statistically significant change in sleeping patterns compared to healthy controls and migraine patients without interictal photophobia [23]. Researchers attributed these findings to the concurrent symptoms of depression and anxiety, commonly associated with migraine [23], suggesting that generalized photophobia might have a stronger relationship with sleep than interictal photophobia, but that mood disorders could be confounders that need to be taken into account.

Light, or rather the lack of light, may be important in migraine patients with photophobia. During a migraine attack, a common behavioral intervention is to seek the comfort of darkness. In one large survey, more than 92% of 2735 participants reported using a dark room during a migraine attack and more than 83% reported it as a consistent strategy to treat their pain [24]. Therefore, it is likely that individuals with migraine with photophobia may experience lower levels of daily light exposure. Given the non-image forming effects of light on circadian rhythms and sleep, we hypothesize that lack of exposure to proper lighting could be contributing to or exacerbating the sleep disturbance experienced by individuals with migraine who have photophobia. However, current literature lacks research that fully investigates the relationship between photophobia in migraine and prevalence of sleep disturbance and poor sleep quality.

In this study, we investigated the relationship between photophobia and sleep (poor sleep quality and sleep disturbance) in individuals with migraine. We hypothesized that generalized photophobia would be associated with poor sleep quality and sleep disturbances in people with migraine even after accounting for the effects of headache frequency, depression, and anxiety.

## Methods

We conducted a cross-sectional observational analysis using existing data extracted from the American Registry for Migraine Research (ARMR)—a multicenter, prospective, longitudinal patient registry, biorepository, and neuroimaging repository [7, 9]. ARMR systematically collected data from individuals experiencing primary and secondary headaches as classified by the ICHD 3 [7]. Institutional Review Boards (IRB) from each of the enrolling sites approved ARMR and all research participants completed and signed an informed consent document. ARMR includes information related to patients’ demographics and types of headaches diagnosed by headache specialists at the medical facilities where they enrolled. Participants completed a set of baseline questionnaires on headache features, personal medical history, sleep quality, anxiety, depression, and photosensitivity, among others. A detailed description of ARMR methods and baseline data have been previously published [7].

### Research participants

Patients included within this analysis of ARMR data were recruited from specialty headache clinics at Mayo Clinic Arizona, University of Utah, University of Texas Health Science Center at Houston, University of Colorado, Georgetown University Medical Center, DENT Neurologic Institute, Dartmouth-Hitchcock Medical Center, Thomas Jefferson University Headache Center, and Yale University. Inclusion criteria for the analysis presented in this study were a clinician diagnosis of migraine according to ICHD-3 diagnostic criteria and completion of the baseline questionnaires by the time of data extraction in July 2023. Headache frequency subtypes were determined according to a participant’s current monthly headache frequency; those reporting 15 or more headache days per month were considered to be in a chronic migraine pattern, while those reporting 14 or fewer headache days per month were considered to be in an episodic migraine pattern. Of note, some participants classified as being in an episodic migraine pattern could have a history of chronic migraine but were in an episodic migraine pattern due to migraine preventive treatment.

The research was performed in accordance with the Declaration of Helsinki and was approved by the Mayo Clinic Institutional Review Board as well as the Institutional Review Boards of each enrolling site mentioned above. All research participants completed and signed an informed consent document.

### Study questionnaires

**Photosensitivity.** Photosensitivity was evaluated using the Photosensitivity Assessment Questionnaire (PAQ) [25, 26]. The PAQ is a self-reported, validated questionnaire that examines behavioral sensitivity to light and psychopathological traits. The questionnaire includes 16 items specifically designed to assess two distinct aspects: “light-avoiding” behaviors associated with photophobia (8 questions) and “light-seeking” behaviors related to photophilia (8 questions). The PAQ utilizes a dichotomous response format, where participants are presented with “Yes” or “No” options to answer each question. Negative answers are rated “0” and affirmative answers are rated “1” for a total score between 0 and 8 for each of the photophobia and photophilia scales. A third score, the global photosensitivity score is obtained through taking the difference between the photophilia and photophobia scores and dividing it by 8. This global score ranges between − 1 (extreme photophobia) and 1 (extreme photophilia) [27]. In this study, we included all 3 scores (photophobia, photophilia, and global photosensitivity scores) in our analysis.

**Sleep.** A self-rated questionnaire was employed in ARMR to assess sleep quality of participants. The questionnaire consists of 50 questions asking participants to provide information on their typical sleep issues as well as their sleep duration, sleep onset latency, and sleep time at night, among others. To determine the prevalence of each sleep issue (i.e., insomnia) in patients with migraine, we analyzed responses to a single self-report question regarding prior diagnoses of any sleep disorder.

To calculate the sleep quality (SQ) in this study, we selected 16 questions that covered sleep disturbance (10 questions), sleep duration (one question), sleep onset latency (one question), use of sleep medication (one question), daytime dysfunctioning (two questions), and subjective sleep quality (one question). Many of these questions were adapted from the Pittsburgh Sleep Quality Index (PSQI) [28], a widely validated questionnaire developed to assess sleep quality and disturbances across diverse populations [29–31]. Although the complete PSQI questionnaire was not included in the ARMR study, supplementary questions were incorporated to evaluate sleep in patients with migraine. The one question for sleep duration asked patients to report their average number of sleep hours during the prior three months while the question on sleep onset latency (SOL) inquired about the number of minutes typically taken to fall asleep during the same period. The questions related to sleep disturbance (SDis) explored the frequency of nighttime or early morning awakenings due to need to use the bathroom, bad dreams, teeth grinding, involuntary limb or body movements, headache or migraine symptoms, stomach pain, non-headache pain, anxiety, depression, and other reasons. Furthermore, the daytime dysfunctioning questions aimed to collect data on participants’ level of enthusiasm and ability to carry out scheduled activities and daily tasks. The one question on sleep medication asked patients on the frequency of taking medications to aid with sleep in the past three months. Lastly, the subjective sleep quality question asked patients to rate the average quality of their sleep in the prior three months. Each question, except for the subjective sleep quality question, was scored on a scale from 0 to 6, where 0 indicated no difficulty or disturbance, and 6 indicated severe difficulty or disturbance. The subjective sleep quality question was scored between 0 and 4, where 0 indicated good quality of sleep, and 4 indicated very poor sleep. The component scores were then summed to generate the total SQ score, which ranged from 0 to 94. Higher scores indicated lower levels of sleep quality and more significant sleep disturbance. While the SDis was included in the overall SQ score, we extracted the score separately for further analysis. A distinct analysis was performed using the SOL reported by patients. We also incorporated an assessment of sleep-related impairment (SRI) in our analysis by including four selected questions that measured daytime sleepiness due to poor sleep quality at night. These questions were adapted from Patient-Reported Outcomes Information System (PROMIS) Sleep Related Impairment Item Bank, a well-validated tool to assess the impacts of sleep disturbances on various aspects of daytime functioning [32–34]. The four selected questions from ARMR asked patients to report any challenges related to waking up in the morning, feeling drowsy upon waking, experiencing daytime sleepiness, and facing daytime issues due to poor sleep at night. Each question was scored on a scale from 0 to 4, with 0 indicating the absence of daytime sleep-related impairment and 4 indicating significant sleep-related impairment. These individual scores were subsequently summed to generate the total score for sleep-related impairment that ranged from 0 to 16. 

**Anxiety.** An evaluation of anxiety symptoms was carried out using the Generalized Anxiety Disorder-7 (GAD-7) assessment tool. The GAD-7 is a short and easy-to-use 7-item scale that has been found to have reliable psychometric properties [35]. The scale examines the frequency of anxiety symptoms experienced by an individual during the preceding two weeks. Response options include “not at all,” “several days,” “more than half the days,” and “nearly daily,” corresponding to scores of 0, 1, 2, and 3, respectively. The scores of 5, 10, and 15 are considered as thresholds for indicating mild, moderate, and severe anxiety, respectively [36].

**Depression.** The Patient Health Questionnaire-2 (PHQ-2) was employed to screen symptoms of depression. The PHQ-2 is a short self-assessment tool consisting of two simple questions that assess the occurrence of depressed mood and anhedonia over the past two weeks. Similar to GAD-7, there are four response options, “not at all,” “several days,” “more than half the days,” and “nearly daily,” which are respectively assigned scores of 0 to 3. The accumulated scores of the two questions yield a total score, ranging from 0 to 6. A score of 3 is established as a cut point for major depressive disorders, whereas a score of 2 suggests potential depressive disorders [37]. The PHQ-2 has been recognized as a valid and reliable screening instrument for identifying depressive disorders in patients with migraine [38].

### Statistics

Data were managed and analyzed using IBM SPSS 28.1 statistical software. In this analysis, the scores from PAQ, the sleep questionnaire, PHQ-2, and GAD-7 were treated as continuous variables due to their extensive distribution covering the full range of possible scores. Descriptive statistics (mean and standard deviation (SD), median (interquartile range), or number (percentage)) were employed to describe the characteristics of sample such as age, sex and race, as well as the prevalence of photophobia, migraine frequency subtype (episodic, chronic), headache frequency, migraine with/without aura, years with migraine, prevalence of sleep problems, depression, and anxiety.

We conducted independent sample t-tests to compare SQ, SDis, SOL, and SRI in patients with and without photophobia. Since insomnia was treated as a categorical variable, Chi-Square was utilized to compare the prevalence of insomnia between patients with and without photophobia. Cohen’s d test was used to find the effect size of photosensitivity on various metrics of sleep.

Separate multiple linear regression models were developed to examine the relationship between the severity of photophobia and SQ, SDis, SOL, and SRI. Similarly, regression models were established to probe the link between photophilia and SQ, SDis, SOL, and SRI. Each regression model generated an R^2^ value as well as a corresponding *P* value for the entire model, alongside individual *P* values for each predictor variable. Diagnostic plots were employed to confirm that all the assumptions for linear regression modeling were met. The SOL data underwent a logarithmic transformation to ensure that the assumption of normality for the linear regression was satisfied. A logistic regression model was developed to describe the relationship between the photophobia scores and the presence of insomnia in patients with migraine.

Regression models were adjusted by accounting for age, sex, and headache frequency per month as covariates. Previous research has highlighted a bidirectional causal relationship between psychiatric symptoms, notably anxiety/depression, and sleep disorders [39–43]. Additionally, a significant correlation has been shown to exist between anxiety/depression and photophobia [23, 44]. In light of these insights, our analysis integrated the scores of anxiety (GAD-7) and depression (PHQ-2) as covariates in the regression models, aiming to isolate the specific influence of photophobia or photophilia on sleep. We also utilized Cohen’s f^2^ to find individual effect sizes of photophobia and photophilia on the sleep-related variables measured in this study. For all analysis, two-tailed hypothesis testing was employed, and statistical significance was set at 95% (*P* < 0.05).

Moreover, a set of moderator analyses was performed using the Process Macro V4.1 [45] in SPSS to explore how migraine subtype (episodic versus chronic) moderates the relationship between photophobia, and photophilia, with sleep-related dependent variables (SQ, SDis, SOL, SRI) included in this study.

We did not conduct a priori power analysis since the sample size was based on available data.

## Results

### Demographics

In total, 852 patients met the inclusion criteria, provided the covariate information, and answered all selected questions related to sleep (Table 1). Patients were 86.6% female (*n* = 738) and 13.4% male (*n* = 114). The mean (SD) age was 49.79 (13.91) years (range = 22–90, *n* = 852). All patients had been diagnosed with migraine, with 62.7% of them classified as being in a chronic migraine pattern (*n* = 534). The mean number of headache days per month for the entire sample was 19.1 (SD = 9.1), while those in a chronic migraine pattern experienced an average of 24 (SD = 6.1) headache days per month, compared to 8.2 (SD = 3.3) for patients in an episodic migraine pattern. The mean duration of living with headaches was 24.1 (SD = 16.6) years. Moreover, 27.1% of patients had a migraine with aura diagnosis (*n* = 231).

### Baseline photophobia and sleep characteristics

The mean score of the PHQ-2 was 1.47 (1.64), and 22.7% of patients (*n* = 193) surpassed the screening cutoff of 2 for potential depressive disorders while 19.8% (*n* = 169) exceeded the cutoff of 3 for major depressive disorder. As for the GAD-7, mean score was 5.87 (5.07), with 8.9% (*n* = 76) of participants meeting the screening threshold for severe anxiety, 10.9% (*n* = 93) for moderate anxiety, and 31.9% (*n* = 272) for at least mild anxiety.

The mean global score of the photosensitivity questionnaire, which does not differentiate between photosensitivity during and between migraine attacks, was 0.11 (0.55) suggesting a photophilic inclination. Totally, 35.6% of patients (*n* = 303) recorded scores below 0, indicating some degree of photophobia. Conversely, about 64.4% (*n* = 549) achieved scores of 0 or above, reporting either no photophobia (6.8%, *n* = 58) or a manifestation of biophilic (light-seeking) behavior (57.6%, *n* = 491).

Roughly 46.5% of patients disagreed with the statement “My overall sleep quality was good,” signifying a subjective perception of poor sleep quality. Insomnia (21.2%, *n* = 181) was the most prevalent sleep complaint reported by patients, followed by obstructive sleep apnea (13.8%, *n* = 118), snoring (10.0%, *n* = 85), bruxism (8.6%, *n* = 73), restless leg syndrome (6.2%, *n* = 53), and nightmares (5.5%, *n* = 48).

**Table 1:** Descriptive statistics summarizing the characteristics of patients and the scores from each questionnaire

Characteristics	Quantity
N	852
Sex, n (%)	Female, 738 (86.6%); Male, 114 (13.4%)
Age in years; mean (SD), range, median	49.79 (13.91), 22–90, 50
Race, n (%)	Asian, 12 (1.4%); Black, 20 (2.3%); White, 802 (94.1%); Native American/Alaskan, 10 (1.2%); Hawaiian/Pacific Islander, 1 (0.1%); not reported, 7 (0.9%)
Years lived with headache, mean (SD)	24.1 (16.6)
Days with headaches per month, mean (SD)	19.1 (9.1)
Chronic Migraine, n (%)*, Average days with headaches per month, mean (SD)	534 (62.7%), 24 (6.1)
Episodic Migraine, n (%)*, Average days with headaches per month, mean (SD)	245 (28.8%), 8.2 (3.3)
Migraine with Aura Diagnosis, n (%)	231 (27.1%)
GAD-7, mean (SD)	5.87 (5.07)
PHQ-2, mean (SD)	1.47 (1.64)
Photosensitivity, mean (SD)	0.11 (0.55)
Light as a headache trigger, n (%)	657 (77%)
Sleep complaints, n (%)	Insomnia, 181 (21.2%), Obstructive sleep apnea 118 (13.8%), Snoring, 85 (10.0%), Bruxism, 73 (8.6%), Restless leg syndrome, 53 (6.2%), Nightmares, 48 (5.6%), Central sleep apnea, 37 (4.3%), Sleep talking, 22 (2.6%), Others < 2%

*n = 73 did not report the number of headaches per month

### Relationship between photophobia and sleep

The mean SQ scores for patients with and without photophobia, determined according to scores on the PAQ, were 31.33 (SD = 14.88, *n* = 303) and 24.33 (SD = 12.80, *n* = 549), respectively. The t-test revealed a statistically significant difference in SQ scores between the two groups (t (850) = -7.17, *p* < 0.001). The effect size (Cohen’s d) was calculated to be 0.513 indicating a medium effect. This suggests that patients with photophobia experience significantly worse sleep quality compared to those without photophobia. Furthermore, the t-test showed a significant difference between patients with and without photophobia in terms of SOL (t (850) = -4.89, *p* < 0.001), SDis (t (850) = -4.70, *p* < 0.001), and SRI (t (810) = -4.28, *p* < 0.001). This indicates that patients with photophobia tend to have longer sleep onset latency and higher levels of sleep disturbances and sleep-related impairments compared to those without photophobia. Table 2 illustrates the means (SD) and results of t-test analysis.

The results of Chi-Square analysis revealed a significant difference between the prevalence of insomnia in individuals with and without photophobia (χ² (1) = 12.06, *p* < 0.001). Patients with photophobia exhibited a higher prevalence of insomnia (30.9%) compared to those without photophobia (19.8%).

**Table 2:** Comparison of SQ, SDis, SOL, and SRI between migraine patients with and without photophobia

Variables	Migraine without photophobia^1^ (*n* = 549)	Migraine with photophobia^2^ (*n* = 303)			
	Mean (SD)	Mean (SD)	t (df)	Cohen’s d	*p*-value
Sleep quality score (SQ)^3^	24.33 (12.80)	31.30 (14.88)	-7.17 (850)	0.51^b^	< 0.001*
Sleep disturbance score (SDis)^3^	13.89 (8.77)	17.04 (10.39)	-4.702 (850)	0.34^a^	< 0.001*
Sleep onset latency (SOL) in minutes	32.65 (30.96)	46.00 (48.46)	-4.89 (850)	0.35 ^a^	< 0.001*
Sleep-related impairment score (SRI)^3^	10.15 (3.81)	11.33 (3.74)	-4.28 (810)	0.31 ^a^	< 0.001*

*Two-tailed, significant at α = 0.05

^a^Cohen’s d calculation: small to medium effect size

^b^Cohen’s d calculation: medium to large effect size

^1^Photosensitivity global score ≧ 0

^2^Photosensitivity global score < 0

^3^Higher scores indicate worse sleep quality, sleep disturbance, and sleep related impairment

The overall regression model for sleep quality (SQ scores), including the primary predictor of photophobia and the covariates, reached statistical significance (Adjusted R^2^ = 0.365, F (6, 772) = 73.851, *p* < 0.001). After controlling for age, sex, headache frequency, depression, and anxiety, the regression coefficient for photophobia was statistically significant (β = 0.157, t = 4.768, *p* < 0.001). This means that for every point increase in photophobia, the sleep quality (SQ score) worsens by 0.157 points. Additionally, there was a statistically significant association between photophobia with SOL (β = 0.096, t = 2.547, *p* = 0.011), SDis (β = 0.115, t = 3.406, *p* < 0.001), and SRI (β = 0.080, t = 2.327, *p* = 0.020), when considering age, sex, headache frequency, depression, and anxiety as covariates in the models. This suggests that higher photophobia scores significantly predict longer sleep onset latency, and increased levels of sleep disturbances and sleep-related impairments in patients with migraine. Table 3 shows results for the photophobia regression analyses.

The logistic regression analysis examining the association between photophobia scores and insomnia presence exhibited statistical significance (Nagelkerke R^2^ = 0.082, *p* < 0.001). After controlling for age, sex, headache frequency, depression, and anxiety, photophobia scores were found to be a significant predictor of insomnia presence, with a positive coefficient (β = 0.110, SE = 0.039, *p* = 0.005), indicating that individuals experiencing photophobia were more likely to have insomnia compared to those without photophobia (Table 4).

Similarly, we conducted a linear regression to investigate the relationship between photophilia and sleep quality in patients with migraine. The overall regression model for sleep quality (SQ scores), with the primary predictor of photophilia and the covariates, reached statistical significance (Adjusted R^2^ = 0.347, F (6, 772) = 69.920, *p* < 0.001). After controlling for age, sex, headache frequency, depression, and anxiety, the regression coefficient for photophilia was statistically significant (β = -0.082, t = -2.702, *p* = 0.007). This indicates that for every point increase in photophilia, the sleep quality improves by 0.082 points. Furthermore, there was a statistically significant association between photophilia with SOL (β = -0.093, t = -2.582, *p* = 0.010), indicating that higher photophilia scores predict shorter sleep onset latency in patients with migraine. We also conducted a separate linear regression to investigate the relationship between photophilia and SRI and SDis with the same covariates. While the overall model was statically significant (Table 3), the β coefficient did not reach statistical significance (SDis: β = -0.52, t = -1.604, *p* = 0.109, SRI: β = 0.006, t = 0.183, *p* = 0.855), suggesting that photophilia, when considered alongside the age, sex, headache frequency, depression, and anxiety, did not have a statistically significant linear relationship with sleep disturbance and sleep-related impairments.

**Table 3:** Linear regressions for investigating relationships between photophobia and photophilia with SQ, SDist, SOL, and SRI

Variable	Regression ^a^				Coefficients		
	Adj. R^2^	F	*p*-value	f^2^	β	SE	*p*-value
**Sleep quality (SQ)**							
Photophobia	0.37	73.85	< 0.001*	0.59 ^d^	0.15	0.16	< 0.001*
Photophilia	0.35	69.92	< 0.001*	0.54 ^d^	-0.08	0.17	0.007*
**Sleep disturbance (SDis)**							
Photophobia	0.25	43.84	< 0.001*	0.34 ^c^	0.12	0.13	< 0.001*
Photophilia	0.24	41.85	< 0.001*	0.32 ^c^	-0.05	0.13	0.109
**Sleep onset latency (SOL)**							
Photophobia	0.08	12.41	< 0.001*	0.09 ^b^	0.10	0.01	0.011*
Photophilia	0.07	10.94	< 0.001*	0.08 ^b^	-0.09	0.01	0.010*
**Sleep-related Impairment (SRI)**							
Photophobia	0.25	41.93	< 0.001*	0.33 ^c^	0.08	0.05	0.020*
Photophilia	0.24	40.73	< 0.001*	0.32 ^c^	0.01	0.05	0.855

*Significant at α = 0.05

^a^ Overall regression model including photophobia or photophilia as the primary predictor and age, sex, frequency of headaches, anxiety, and depression as covariates

^b^f^2^ = Cohen’s F calculation: small effect size

^c^f^2^ = Cohen’s F calculation: small to medium effect size

^d^f^2^ = Cohen’s F calculation: medium to large effect size

SE = Coefficients Standard Error

*n* = 779

Moreover, the logistic regression analysis investigating the association between photophilia scores and the presence of insomnia demonstrated a statistical significance (Nagelkerke R^2^ = 0.078, *p* < 0.001). After controlling for age, sex, headache frequency, depression, and anxiety, it was observed that photophilia scores emerged as a significant predictor of insomnia presence, with a negative coefficient (β = -0.095, SE = 0.039, *p* = 0.014), indicating that higher photophilia scores are associated with a decreased likelihood of insomnia among patients with migraine (Table 4).

**Table 4:** Logistic regressions for investigating relationships between photophobia and photophilia with insomnia

Variable	Regression ^a^		Coefficients			
	Nag. R^2^	*p*-value	β	SE	*p*-value	Unit odds ratio
**Insomnia**						
Photophobia	0.08	< 0.001*	0.11^b^	0.04	0.005*	1.17
Photophilia	0.08	< 0.001*	-0.10 ^b^	0.04	0.014*	0.91

*Significant at α = 0.05

Nag. R^2^ = Nagelkerke R Square

^a^ Overall regression model including photophobia or photophilia as the primary predictor and age, sex, frequency of headache, anxiety, and depression as covariates

SE = Coefficients Standard Error

*n* = 704

Our moderator analyses revealed no significant moderating effects of migraine subtype (chronic versus episodic) on the relationship of photophobia and photophilia, with sleep quality, sleep onset latency, sleep disturbances, sleep-related impairments, and insomnia. Table 5 illustrates the results for each variable. These findings suggest that the impacts of photophobia and photophilia on the sleep outcomes examined in this study remain consistent across different migraine frequency subtypes. All the models were adjusted for age, sex, depression, and anxiety.

**Table 5:** Moderator analyses: the effects of migraine subtype on photophobia and photophilia relationship with sleep outcomes

Variable	Photophobia x Migraine type				Photophilia x Migraine type			
	β	SE	t	p-value	β	SE	t	p-value
Sleep quality (SQ)	-0.15	0.30	-0.49	0.623	0.04	0.33	0.12	0.901
Sleep disturbance (SDis)	-0.04	0.24	-0.16	0.875	0.12	0.26	0.46	0.644
Sleep onset latency (SOL)	0.004	0.01	0.40	0.691	-0.02	0.01	-1.43	0.155
Sleep-related Impairment (SRI)	-0.05	0.10	-0.52	0.603	-0.15	-0.11	-1.43	0.154
Insomnia	0.01	0.07	0.21	0.835	-0.04	0.07	-0.60	0.370

## Discussion

We investigated the relationship between photosensitivity and various metrics of sleep including sleep quality, sleep disturbance, sleep onset latency, sleep-related impairments, and insomnia in patients with migraine. The results were obtained using existing data extracted from ARMR from 852 patients who met the inclusion criteria. Poor sleep quality was prevalent among our participants, with over 46% reporting lack of good sleep quality. In line with several previous inquiries [15, 46], our data exhibited insomnia as the most frequently reported sleep disorder among patients with migraine. Additionally, this study presents novel findings, demonstrating that patients with migraine with generalized photophobia tend to have poorer sleep quality compared to those without photophobia (including patients with photophilia). Both photophobia and photophilia exhibited statistically significant associations with sleep quality, sleep onset latency, and insomnia. As we hypothesized, greater severity of photophobia emerged as a predictor for compromised sleep quality, increased sleep disturbance, prolonged sleep onset latency, and higher likelihood of insomnia presence. Conversely, a tendency for light, or photophilia, was correlated with enhanced sleep quality, shorter sleep onset latency, and a reduced likelihood of insomnia. These results persisted after controlling for patients’ age, sex, headache frequency, anxiety, and depression. Moreover, the relationship between photophobia and photophilia with sleep-related outcomes examined in this study remained consistent regardless of migraine frequency subtype, whether chronic or episodic. Our findings are aligned with a limited number of prior studies that reported an association between photophobia and sleep disorders in migraine [22, 23] as well as with the emerging body of literature highlighting the complex effects of light exposure on sleep quality [47–49].

Prior studies have demonstrated that retinal light exposure serves as a direct stimulant for the brain’s biological clock, exerting profound effects on various facets of sleep such as sleep pattern, sleep onset latency, sleep duration, and sleep efficiency [50–52]. The rhythmic interplay of light and darkness experienced throughout the day intricately orchestrates the timing of the circadian clock. To synchronize harmoniously with the natural light-dark cycle of Earth, a consistent exposure to high levels of morning light and a contrast between daytime and nighttime light exposure are essential. In a study by Figueiro et al. [53], it was observed that exposure to high levels of lighting in the morning was associated with reduced sleep onset latency, increased phasor magnitudes (which is a measure of circadian entrainment), and increased sleep quality in adult participants. These findings were aligned with other similar inquiries on other age groups [54–56]. Moreover, research showed that the human circadian system and ipRGCs in retina exhibit heightened sensitivity in the blue portion of the light spectrum, particularly around 479 nm [57, 58]. Thus, blue wavelengths have been demonstrated to have a more powerful impact on the human circadian rhythm compared to wavelengths in the green, yellow, and red regions [59]. Studies have reported that exposure to blue-enriched white light during daytime hours can enhance sleep quality, extend the duration of nocturnal sleep, and diminish sleep disturbances across diverse populations [49, 60–63]. Whereas there is growing evidence for circadian relevance in migraine and other pain disorders [21, 64], none of the previous studies were focused on circadian effects of light on patients with migraine with photosensitivity.

A prevalent coping strategy to alleviate discomfort and pain induced by migraine-associated photosensitivity involves seeking refuge in darkness, often by spending extended periods in dimly lit or dark rooms and donning dark or tinted sunglasses. Additionally, those with migraine with photosensitivity exhibited lower discomfort thresholds when exposed to blue wavelengths of light in comparison to green light [65]. These behaviors not only reduce the retinal exposure to circadian effective light during the daytime in patients with migraine, but also inadvertently result in constant lighting conditions, devoid of the natural contrast between the day and night. Consequently, the biological clock is deprived of a crucial time cue to stimulate the circadian clock accordingly, potentially leading to disruptions in sleep, prolonged sleep onset, and poor sleep quality.

In those with migraine, insufficient sleep and sleep disturbance have consistently emerged as frequent headache triggers [12, 66–68]. Conversely, adequate sleep timing, duration, and quality has been reported to be protective against headache [69]. Given the therapeutic effects of high-quality sleep in managing migraine and the potential adverse effects of pharmacological treatments, implementing non-drug interventions appears to be an effective initial approach to improve sleep quality in this population. Lighting, in particular, has been demonstrated as a potent non-pharmacological intervention capable of improving sleep and circadian activity rhythms, among other benefits [47–49, 70, 71]. In a study by Burgess et al. [72], the findings revealed that bright light therapy in the morning advanced circadian phase which was associated with an increased tolerance to pain. The positive association we found between photophilia and improved sleep outcomes further supports the potential beneficial effects of light exposure on sleep outcomes amongst those with migraine. It is reasonable to assume that patients with migraine with photophilia tend to receive more retinal light exposure during the daytime which could potentially be the reason for enhanced sleep outcomes in this population. In a recent open-label study, self-reported data revealed that two hours of exposure to narrow band green light led to an improvement in photophobia and same-night sleep for 53% and 49% of all migraine attacks, on average, respectively [18]. However, it is not clear whether the improvement in sleep was due to the effects of light exposure on circadian activity rhythms or as a result of enhanced mood, photophobia, or headache improvement.

Circadian stimulation by light is achieved through exposure to high-intensity lighting with a blue-enriched spectrum during the daytime. Nevertheless, the prevalence of photophobia in migraine poses a challenge to the implementation of circadian lighting for this population, given their severe sensitivity to high intensity light, particularly in the blue spectrum [65, 73]. The emergence of new lighting technology holds promise for enhancing well-being among those with migraine through offering customized lighting solutions with optimized intensity and spectrum tailored to individuals’ needs, while maintaining circadian rhythms by following a natural light/dark cycle. Exposure to proper circadian lighting not only has the potential to improve sleep quality in patients with migraine but also could contribute to positive effects on anxiety and depressive symptoms – both highly associated with migraine and photophobia.

The recruitment of patients from nine different sites across the United States, the large sample size, and the diverse geographical locations of enrollment enhance the potential generalizability of our findings. However, the findings of this study are limited as we only included patients from headache specialty centers who tended to have high frequency migraine and likely represent a population with severe migraine disease. To improve generalizability, future studies should include a more diverse population. Our study is also limited by its reliance on self-reported data for photosensitivity and sleep quality, collected through instruments selected by the ARMR study team. Self-reported data are inherently subjective as they rely on individuals’ subjective perceptions, introducing the possibility of recall bias. Additionally, the photosensitivity questionnaire (PAQ) utilized in ARMR, while well-validated, lacks specific assessments of headache-related light sensitivity, focusing instead on non-specific behavioral preferences. Similar limitations apply to the sleep questionnaire, which incorporates partial components from various instruments and does not include the entire set of questions from a single validated questionnaire. That makes interpretation and analysis of the sleep data challenging. Future studies should consider supplementing subjective measures with objective ones (i.e., actigraphy, wearable light trackers) to acquire precise data on sleep quality and daily light exposure behavior among patients with migraine. Furthermore, experimental trials aimed at investigating the effects of various lighting interventions on sleep quality and circadian health of those with migraine could offer valuable insights. Such research might pave the way for the development of novel lighting technologies, contributing to the improvement of well-being in this population.

Furthermore, while our study examined photosensitivity in relation to sleep among patients with migraine, we recognize the existence of various other sensory sensitivities (i.e., phonophobia, osmophobia, tactile sensitivity) that may exert similar influences on sleep quality and disturbances in this population. Future studies should explore the intricate association between each of these heightened sensitivities and their impact on sleep-related outcomes in migraine. Moreover, examining the potential interplay among these sensory sensitivities can provide a more meticulous understanding of the multifaceted risk factors contributing to sleep disturbances in migraine.

Lastly, it is important to acknowledge a limitation inherent in the ARMR dataset [7], which pertains to the unbalanced racial distribution of the sample. The participants in this registry are predominantly White, a characteristic that is apparent in the sample included in our analysis (94.1% White). This feature of the dataset, undeniably, limits the generalizability of our findings. It is imperative for future research to include a more diverse sample for extrapolating the results.

## Conclusion

This study demonstrated that patients with migraine with photophobia experienced worse sleep quality compared to those without generalized photophobia. Both photophobia and photophilia exhibited significant associations with sleep quality, insomnia, and sleep onset latency, persisting after adjusting for age, sex, headache frequency, anxiety, and depression. This relationship could be explained by the well-established influence of light on circadian activity rhythms mediated by the ipRGCs in the retina. Given the high prevalence of photophobia and sleep disorders among patients with migraine and their impacts on overall quality of life, these findings highlight the need for in-depth systematic exploration of the relationship between photophobia and sleep in this population. Such research can provide insights that will help pave the path for developing novel lighting technologies and design solutions to address the specific needs of those with migraine.

## Data Availability

The datasets analyzed in this study are contained within the American Registry for Migraine Research (ARMR). Data access requests should be directed to the American Migraine Foundation.
